# Suicide Attempts in Ilam Province, West of Iran, 2010-2014: A Time Trend Study

**Published:** 2016-05-08

**Authors:** Yousef Veisani, Ali Delpisheh, Kourosh Sayehmiri, Ghobad Moradi, Jafar Hassanzadeh

**Affiliations:** ^a^ Psychosocial Injuries Research Center, Ilam University of Medical Sciences, Ilam, Iran; ^b^ Department of Clinical Epidemiology, School of Public Health, Ilam University of Medical Sciences, Ilam, Iran; ^c^ Department of Social Medicine, School of Medicine, Ilam University of Medical sciences, Ilam, Iran; ^d^ Social Determinants of Health Research Center, Kurdistan University of Medical Sciences, Sanandaj, Iran; ^e^ Research Center for Health Sciences, Department of Epidemiology, School of Health, Shiraz University of Medical Sciences, Shiraz, Iran.

**Keywords:** Suicide attempt, Suicide, Iran

## Abstract

**Background:** Suicide has become an increasingly widespread form of morbidity in the developing countries. There has been an increasing trend in morbidity and mortality due to suicide in Iran over the past few decades. This study surveyed attempts and completed suicide over a 5-year period.

**Methods:** Through a cross-sectional study, overall identified suicides by systematic registration suicide data (SRSD) in Ilam Province, western Iran from 21 March 2010 to 11 December 2014 were enrolled. Multiple logistic regression analysis was used for measuring the association between the risk factors of interest and suicide. The statistical software package was Stata 11.2.

**Results:** A Suicide attempts have slightly increased in Ilam during 2010-2014, during which, 6,818 attempted suicides occurred of which 546 were completed. The odds of completed suicide was higher among older age groups than younger ones so that the crude OR estimates of completed suicide among people aged 50 to 59 yr against people aged <20 yr was (OR=6.99; 95% CI: 3.02, 11.07). The crude and adjusted odds ratio (OR) estimates of completed suicide in males against females were (OR=3.22; 95% CI: 2.58, 3.93) and (OR=3.66; 95% CI: 3.03, 4.11), respectively. Significant excess risk also appeared with academic against illiterate attempters (OR=2.31; 95% CI: 1.35, 3.95). Results showed no increasing trend in the suicide method. Some methods such as self-immolation had decreasing trend over time, although it was not statistically significant (*P*=0.089).

**Conclusions:** We observed the variety of suicide risk factors that calls for more diversity in preventative programs. Distribution of suicide methods is diverse across the period of the study.

## Introduction

Suicide is one of the ten leading causes of death in Western countries, and the second leading cause for individuals between the ages of 15 and 19 yr^1^. In recent decades, annual parasuicide have reportedly ranged from 2.6 to 1,100 per 100,000 individuals, while the lifetime prevalence rate ranges from 750 to 5,930 per 100,000 worldwide^2^. Not surprisingly, 84% of all emerging cases will do so in low- and middle-income developing countries^3^. A 2014 systematic review of the Eastern Mediterranean Region by WHO reported that the incidence of committed suicide ranged from 0.55 to 5.4 per 100,000^4^ and epidemiological studies have investigated geographical variations in suicide in an effort to identify additional explanatory variables^5-7^.

In a resent systematic review, that studied social related factors affected to suicide among Iranian population family conflict (32%), marital problems (26%), economic constrains (12%), and educational failures (5%) were the most frequent cause of attempted suicide^8^. Some previous province-level studies^9-12^ have shown that adults aged 20-29 yr, familial problems, psychiatric disorders, females, the married, high school graduates, and housewives, history of previous suicide attempt were the significant factors regarding to suicide.

Developing countries due to a number of reasons (e.g. lack of preventive programs and less reliable existing data, etc.) are less contributed to the international knowledge production regarding suicide. This is more apparent, especially in determining trend of different methods of suicide.

Based on our knowledge Ilam has a first rank of suicide rate (19.45 per 100000 people) among 31 provinces in Iran^13^.

The current study with representative and fresh data aimed to examine the association of attempter characteristic and suicide. As the second objective, we also determined the trend of incidence and suicide methods.

## Methods

**Figure 1:**
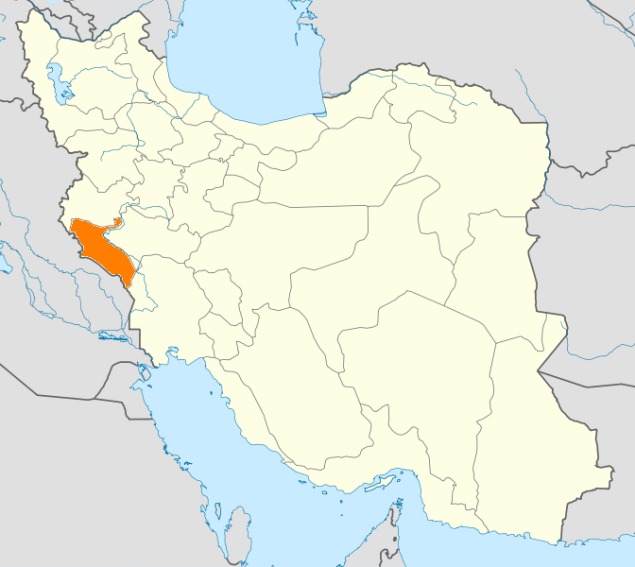
The geographical location of Ilam Province in Iran

Data of suicide attempts were extracted from the systematic registration suicide data (SRSD) system provided by Ilam University of Medical Sciences, Ilam Province (Figure 1). Data on attempted suicide have been collected systematically since 2010. Suicide attempt was defined as cases in which the response indicated suicide and completed suicide was defined as cases in which they died from suicide.

This study recognized 546 suicide deaths and 6818 attempted suicides from 21 March 2010 and 11 December 2014. The data for the present study consisted of all suicide attempts in the Ilam Province by residents ages six years and older who had admitted to healthcare during the period of study. We used the data on population from Statistical Center of Iran published in 2011 and the subsequent estimates for the following years till 2014 to estimate of annual suicide incidence rate per 100,000. In SRSD, suicide attempts were determined through the analysis of physician claims and hospital admission records, in addition to daily suicide counts according to a structured schedule which involved nine items: age, sex, marital status, educational level, job status, partners’ job status and educational level, region of residence, race, and other demographic information. Data concerning mental disorders, addiction, methods of suicide attempt, and outcome were collected from individual outpatient visits on a monthly basis. Besides, SRSD updated monthly using data from the Center of Province Forensic Medicine to confirm and compare a completed suicide cases. Multiple logistic regression analysis was used for measuring the association between the risk factors of interest and suicide. All analyses were performed at 0.05 significant levels using SPSS ver. 11.2 (Chicago, IL, USA).

## Results

During the five-year study period, 6,818 attempted suicides occurred of which 546 were completed. as 0.08 (95% CI: 0.073, 0.086). Death following attempts in rural and urban area was 0.10(CI95%: 0.092-0.122) and 0.07 (95% CI: 0.064, 0.078), respectively which represents use of violent suicide methods in rural areas. Completed suicide for male and female was 0.08 (95% CI: 0.071, 0.090) and 0.07 (95% CI: 0.070, 0.088), respectively. According to Table 1, the proportion of females was 53.7%, 55.5% of the study sample entailed people between aged 16-25 yr; in the hole of the participants 56.9% were single; the majority of them allotted to residence to rural area 76.1%, the housewife job 32.6%, and had a diploma education level 40%.

**Table 1:** General characteristics of the study population using multiple logistic regression analysis

**Variables**	**Suicide attempt** **(n=546)**	**Suicide death** **(n=6818)**	**Unadjusted** **OR** **(95% CI)**	***P*** ** value**	**Adjusted** **OR** **(95% CI)**	***P*** ** value**
**Age group (yr)**						
<20	9	156	1.00		1.00	
20-29	299	3782	1.78 (1.02, 3.31)	0.001	1.66 (0.88, 2.55)	0.001
30-39	186	2158	2.35 (1.42, 5.09)	0.001	2.22 (1.30, 4.77)	0.001
40-49	49	415	4.23 (2.14, 7.88)	0.001	3.09 (1.95, 6.93)	0.001
50-59	45	178	6.99 (3.02, 11.77)	0.001	6.08 (2.77, 9.63)	0.001
≥60	28	102	7.56 (4,03, 13.45)	0.001	6.33 (3,85, 11.91)	0.001
**Gender**						
Female	3661	291	1.00		1.00	
Male	3157	255	3.22(2.58, 3.93)	0.001	3.66 (3.03, 4.11)	0.001
**Occupation**						
Housewife	190	2220	1.00		1.00	
Unemployment	186	2143	0.95 (0.33, 1.89)	0.088	1.58 (1.12, 2.30)	0.001
Student	42	1188	1.40 (0.93, 1.87)	0.007	1.70 (1.07, 2.73)	0.075
Employed ^a^	107	1005	0.44 (0.23, 1.02)	0.110	0.88 (0.43, 1.23)	0.093
Others ^b^	21	262	1.03 (0.88, 1.33)	0.003	1.26 (0.95, 1.56)	0.001
**Educational level**						
Illiterate	773	133	1.00		1.00	
Primary school	401	82	0.66 (0.47, 0.93)	0.007	2.03 (1.42, 2.06)	0.001
Guidance/high school	1988	157	1.64 (1.17, 2.28)	0.001	1.92 (1.36, 2.50)	0.001
Diploma	2775	136	2.67 (1.85, 3.87)	0.001	3.30 (2.39, 5.06)	0.001
University	881	38	2.31 (1.35, 3.59)	0.001	2.98 (1.77, 4.39)	0.001
**Marital statues**						
Marriage	289	2937	1.00		1.00	
Single	257	3881	0.77 (0.57, 1.03)	0.003	1.62 (1.22, 2.06)	0.001

^a^ Employed included employee and free jobs ^b^Others included soldiers, retired, workers, etc.

Logistic regression was used to examination risk of the variables to completed suicide. The odds of completed suicide was higher among married individuals compared to single people (OR=1.62; 95% CI: 1.22, 2.06). The crude and adjusted odds ratio (OR) estimates of completed suicide in males against females were (OR=3.22; 95% CI: 2.58, 3.93) and (OR=3.66, 95% CI: 3.03, 4.11) respectively. Significant excess risk also appeared with academic against illiterate attempters (OR=2.31, 95% CI: 1.35, 3.95). Compared with housewife, student attempters were significantly related to a higher risk of completed suicide (OR=1.40; 95% CI: 0.93, 1.87). The odds of completed suicide was higher among older age groups than younger ones so that the crude OR estimate of completed suicide among people aged 50 to 59 yr against people aged <20 yr was (OR=6.99; 95% CI: 3.02, 11.07) (Table 1).

The distribution of absolute number of suicides and the incidence rate per 100000 of attempted and completed suicide is given in Table 2. The incidence rates of attempted suicide have increased in recent years, although the incidence rates of completed suicide were not changed significantly. As a whole, suicide attempts have increased from 2010 to 2012 and then dropped, but in 2014 compared to 2010 was higher.

**Table 2:** Incidence rate of suicide attempt and completed suicide per 100,000 and by year

**Year**	**Population**	**Frequency of suicide**		**Incidence rate of suicide per 100,000**	
		**Attempt**	**Death**	**Attempt**	**Death**
2010	533623	961	196	180	33.9
2011	557599	1185	94	212	16.8
2012	581575	1610	128	276	22.0
2013	606582	1359	82	224	13.5
2014	632665	1203	46	190	7.3

**Figure 2:**
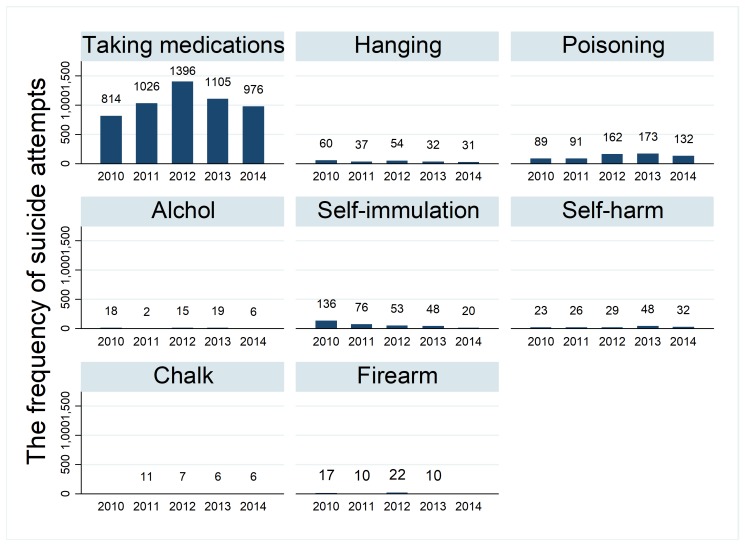
Distribution plot of suicide methods, stratified by year of study

Analysis of data revealed that taking medications (78.0%) and self-poisoning (9.5) were the most common methods for suicide. Other common means included self-immolation (4.9), hanging (3.1%), self-harm (2.3%), alcohol ingestion (0.9%), firearm (0.9%), and chalk ingestion (0.4%). Taking medications accounted about 87.6% of all non-violent suicides among males and over 88% of female non-violent suicides. Nonetheless, only 2% of those who had attempted suicide by taking medications had eventually died. In other methods, 44% of those who used a self-immolation, 38% of those who poisoned themselves with chalk, and 31% of those who hanged themselves had died (Figure 2). Results showed no increasing trend on suicide method. Some methods such as self-immolation had decreasing trend over time, although it was not statistically significant (0.089).

## Discussion

A review of the extant literature revealed disparities between recorded suicide rates in Ilam Province and national reports from Iran (14). Western provinces including Ilam, Kermanshah, and Hamedan had the highest rate of suicide in Iran, including 20, 14, and 10, respectively, per 100,000 people between 2006 and 2010 ^13-14^. In addition, suicide rates in recent years (2006–2010) reported a higher annual percentage change (APC) in Ilam (+10%) compared to other provinces^13^. Based on the SRSD, the overall suicide rate in Ilam was 19.54 per 100,000 persons between the years 2010 and 2014, possible explanations for which include low economic development, extensive regional war zones (1980- 1986), and dominance of traditional culture ^15^.

Suicide has been linked to multiple risk factors, including biological, mental health, and social factors^16^. Understand the factors associated to attempts and completed suicide is fundamental for the purposes of suicide prevention. In logistic regression analysis the findings regarding the effect of residence, educational level, and mental disorders on suicide (with adjustment only for age and gender) are compatible with other reports ^17-19^. As for other risk factors, single people, age, educational level‏ and substance and alcohol use disorder are more likely to commit suicide ^20-24^, consistent with our study that shows a significantly higher risk of suicide for single attempter’s people with college degrees, and in males.

Over 12% of both genders who committed suicide in Ilam during 2010–2014 choose a violent method. Hanging was the majority of violent methods among males and self-immolation was common means in females. Taking medications was the most common means for non-violent suicide in both genders. When compared to other national reports, the attempters in Ilam seem to use more violent methods in committing suicide. A similar percentage in violent suicide means for males and females in Sari was reported consistent with our study^11^.

The present study used SRSD, Ilam Province, Western Iran for the time period 2010–2014. Using the large sample attempters, the present study was able to secure representativeness. The present study suffered several limitations, including data reliability, underreporting, and generalizability. As a result of using self-reported data, we ran the risk of introducing bias into our analysis. In addition, the SRSD is often assumed to fall victim to underreporting; therefore, the proposed suicide rate is potentially underestimated in this study^21^. Finally, while the SRSD system is available in the provincial capital of Ilam, other cities of the province cannot register data directly; therefore, the findings of this study may not be generalizable to larger populations.

## Conclusions

Age, residence, job status, educational level‏ and marital status‏ were significantly associated with completed suicide. Distribution of suicide methods is diverse across the period of the study. We concluded that factors associated with completed suicide vary in Ilam that calls for more diversity in preventative programs.

## Acknowledgments

We would like to thank Vice-Chancellor of Research and Technology of Ilam University of Medical Sciences for financial support of this study.

## Conflict of interest statement

The authors declare that they have no conflicts of interest.
